# Spatial Distribution of Breast Cancer in Morocco and the Impact of Travel Distance and Rural Residence on Cancer Stage

**DOI:** 10.3390/epidemiologia6040080

**Published:** 2025-11-25

**Authors:** Chaimaa Elattabi, Jeroen Berden, Najoua Lamchabbek, Ilhame Bourais, Karima Bendahhou, Saber Boutayeb, Najia Mane, Siham Mrah, Inge Huybrechts, Elodie Faure, Mohamed Khalis

**Affiliations:** 1 Department of Public Health, Mohammed VI Center for Research and Innovation, Rabat 10112, Morocco; 2 International School of Public Health, Mohammed VI University of Sciences and Health, Casablanca 82403, Morocco; 3 Nutrition and Metabolism Branch, International Agency for Research on Cancer, 69366 Lyon, France; 4 Department of Food Technology, Safety and Health, Faculty of Bioscience Engineering, Ghent University, 9000 Ghent, Belgium; 5 Cancer Registry of the Grand Casablanca Region, Casablanca 20000, Morocco; 6 Laboratory of Epidemiology and Research in Health Sciences, Faculty of Medicine, Pharmacy and Dental Medicine, Sidi Mohamed Ben Abdallah University, Fez 30070, Morocco; 7 Laboratory Research of Cancer and Chronic Diseases, Faculty of Medicine and Pharmacy of Tangier, Abdelmalek Essaadi University, Tetouan 93000, Morocco; 8 French Network for Nutrition and Cancer Research (Nacre Network), 78350 Jouy-en-Josas, France; 9 Higher Institute of Nursing Professions and Health Techniques, Ministry of Health and Social Protection, Rabat 10000, Morocco; 10 Center of Epidemiology and Population Health, Paris Saclay University, 94805 Villejuif, France

**Keywords:** cancer diagnosis, geographic accessibility, health disparities, rural health, breast cancer, geographic distribution, health services accessibility, rural vs. urban residence, cancer stage, healthcare access, Geographic Information System

## Abstract

Introduction: Breast cancer is the most common cancer among women worldwide, and its prognosis can be influenced by various factors, including geographic accessibility of healthcare services. This study describes the geographic distribution of breast cancer cases in the Casablanca-Settat region and evaluates the association between breast cancer stage at diagnosis, rural residence, and travel distance to healthcare facilities in the Casablanca-Settatregion. Methods: A retrospective hospital-based study was conducted on 2161 women diagnosed with breast cancer and admitted to Ibn Rochd University Hospital between December 2018 and January 2022. Patient residential addresses and healthcare facility locations were geocoded using Geographic Information Systems (GIS), and a straight-line distance was calculated from patients’ residences to the nearest Primary Healthcare Center (PHCC), Provincial Hospital Center (PHC), Regional Hospital Center (RHC), and University Hospital Center (UHC). Statistical analysis assessed associations between stage at diagnosis, rural/urban residence, and travel distance. Results: The overall mean distance to the UHC was 32.5 km (range: 0.19–164 km); 8.3 km (range: 0.02–83 km) to PHCs; and 1.25 km (range: 0.01–12.1 km) to PHCCs. Rural patients had longer distances to all facility types compared to urban patients. However, no significant association was found between cancer stage at diagnosis and rural residence or long travel distance to healthcare facilities (*p* > 0.05). Conclusions: The stage of breast cancer at diagnosis appears not to be influenced by travel distance to healthcare facilities or by rural residence.

## 1. Introduction

Breast cancer is the most common cancer in women and a major public health concern worldwide [1]. According to GLOBOCAN, breast cancer accounted for 2,308,897 new cases and 665,684 deaths globally in 2020 [2]. In Morocco, breast cancer is the most common cancer among women, with 58.4 new cases per 100,000 women [3]. Despite efforts to improve early detection and treatment, the mortality rate from breast cancer remains significant, with 18.1 deaths per 100,000 women [3]. According to the literature, 20% to 40% of the mortality rate could be prevented if breast cancer cases were diagnosed at an early stage [4,5,6].

Several studies have focused on the determinants of advanced cancer stage. They have notably examined the impact of healthcare infrastructure, early detection, and geographic access on delays in diagnosis and advanced disease stage. Several studies that evaluated the impact of travel distance on cancer stage reported that longer travel distance to healthcare facilities was associated with late cancer stage at diagnosis [7].Similarly, our recent systematic review examining the impact of travel distance on cancer stage found that the association varied depending on the country of study with the majority of studies reporting a positive association being from Africa [8]. Also, studies evaluating the impact of rural residence on cancer stage consistently show that rural women are more likely to be diagnosed with later-stage breast cancer [7,9,10,11,12]. For example, a systematic review and meta-analysis including over 870,000 women showed that rural residents were more likely to be diagnosed at an advanced stage compared with their urban counterparts [13]. Similarly, analyses from the Surveillance, Epidemiology, and End Results Program registry in the United States reported that women living in rural areas had a higher proportion of late-stage breast cancers compared with those in urban areas, even after adjusting for sociodemographic and clinical factors [14].In Morocco, 62.8% of the population lives in urban areas while 37.2% resides in rural areas [15], access to treatment hospitals may vary depending on the place of residence, and public transportation is usually limited in rural areas [16]. Therefore, the impact of travel distance to healthcare facilities needs to be carefully examined as it is likely to play a major role. The Moroccan public health sector, which serves approximately 85% of all patients [17], has a hierarchical referral system [18]. In this sector, a breast cancer patient seeks help in primary healthcare centers after noticing disease symptoms herself. She is then referred to a secondary hospital for diagnosis and subsequently to a university hospital center for treatment. As a result, patients—particularly those living in rural or remote areas—often need to travel considerable distances to access appropriate diagnostic and treatment services. These geographic and logistical barriers can contribute to significant delays in the diagnostic pathway. Despite their potential impact, the effect of travel distance, rural residence, and other access-related challenges on cancer stage at diagnosis remains largely understudied in the Moroccan context.

Although international and African studies suggest that distance to healthcare facilities and rural residence are associated with later-stage breast cancer, evidence from Morocco remains limited. A study conducted at the National Institute of Oncology in Rabat described that travel distances of cancer patients ranged from 5 km to 1200 km, with a median of 118 km, with 68.7% of patients coming from rural areas with geographic barriers and 85.3% reporting the high cost of transport [19]. However, it did not assess their relationship with cancer stage at diagnosis. Moreover, no research to date has mapped the geographic distribution of breast cancer cases or explored the combined influence of travel distance and rural residence on stage at diagnosis in the Casablanca-Settat region, which is the most populated area and home to one of the country’s main university hospital centers.

In this context, our study draws data from 2161 breast cancer cases living in the region of Casablanca-Settat and admitted to the Ibn Rochd University Hospital, with the objectives of mapping the geographic distribution of breast cancer cases across the Casablanca-Settat region and examining the association between advanced cancer stage, rural residence, and travel distance to healthcare facilities. By addressing these questions, our study aims to inform strategies that promote equitable access to care and early diagnosis, ultimately contributing to improved survival outcomes for Moroccan women with breast cancer.

## 2. Materials and Methods

### 2.1. The Study Area

**Figure 1:**
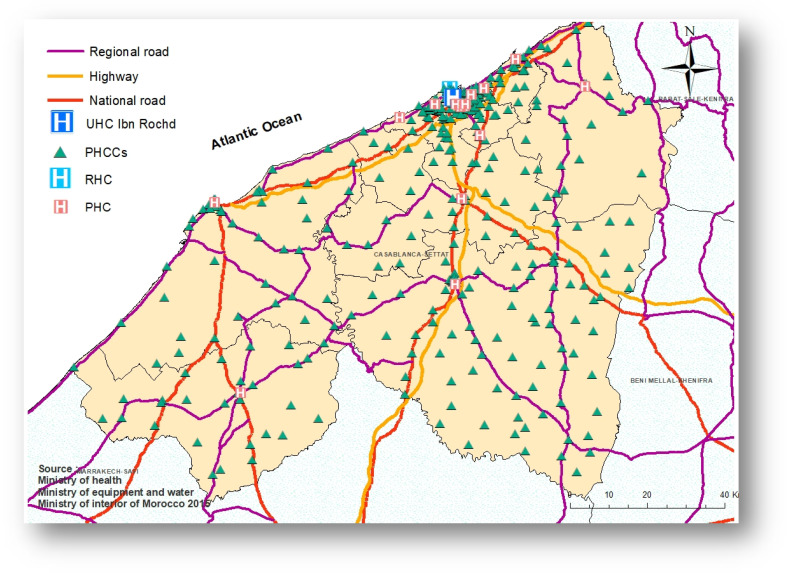
The region of Casablanca-Settat, Morocco.

The study area is the region of Casablanca-Settat, the most populous region in Morocco. This region covers an estimated area of 19,448 square kilometers, accounting for nearly 2.7% of the national territory. According to the General Population and Housing Census of 2014 the Casablanca-Settat region housed 7,408,213 people, accounting for 20.3% of the total population of Morocco. The region is divided into 9 provinces as shown in Figure 1.

### 2.2. Study Period and Data Source

We included women with histologically confirmed breast cancer who were admitted to the Ibn Rochd University Hospital in Casablanca. Data were taken from the ENOVA electronic medical system, where physicians routinely enter patient information after consultation. The study covered the period from December 2018 to 2022. In total, 2161 women were included. However, patients without diagnostic confirmation, those with missing addresses, and patients not residing in the Casablanca-Settat region were excluded from our study. We hypothesize that most of these women were initially diagnosed within the public healthcare system, as the UHC provides free treatment for individuals covered by the RAMED program, which supports economically disadvantaged populations relying on public healthcare services. The study period was selected to coincide with the implementation of the ENOVA electronic data management system at the UHC, which was introduced in 2018, replacing paper-based records.

### 2.3. Data Collection

The extracted data included demographic information (age (years), weight (kg), height(m), BMI (kg/m^2^), type of health coverage, and residential address), key dates (date of diagnosis confirmation, file opening date, and date of treatment initiation, all using the day/month/year format), and tumor characteristics, based on TNM classification [20].The Tumor size, Node involvement, Metastasis(TNM) classification was converted into conventional breast cancer stages to facilitate analysis and interpretation. Each patient’s TNM code was mapped to a corresponding stage according to standard criteria: Stage I for small, localized tumors without lymph node involvement; Stage II for larger tumors or those with limited spread to nearby lymph nodes; Stage III for extensive local disease or significant regional lymph node involvement; and Stage IV for distant metastasis. After assigning stages, cases were further grouped into two main categories for analysis: early-stage cancer (Stages I and II) and advanced-stage cancer (Stages III and IV) [9]. Health insurance coverage was classified into three categories:(1) RAMED, Morocco’s program offering free healthcare services to vulnerable populations relying on public healthcare; (2) public health insurance (CNOPS), coverage for civil servants and employees in the public sector; and (3) private insurance (CNSS, SAHAM, etc.), coverage for private-sector employees with both of health coverages covering oncology services in public and private sectors.

### 2.4. Geographical Data, GeocodingProcess, and Travel Distance Calculation

**Figure 2:**
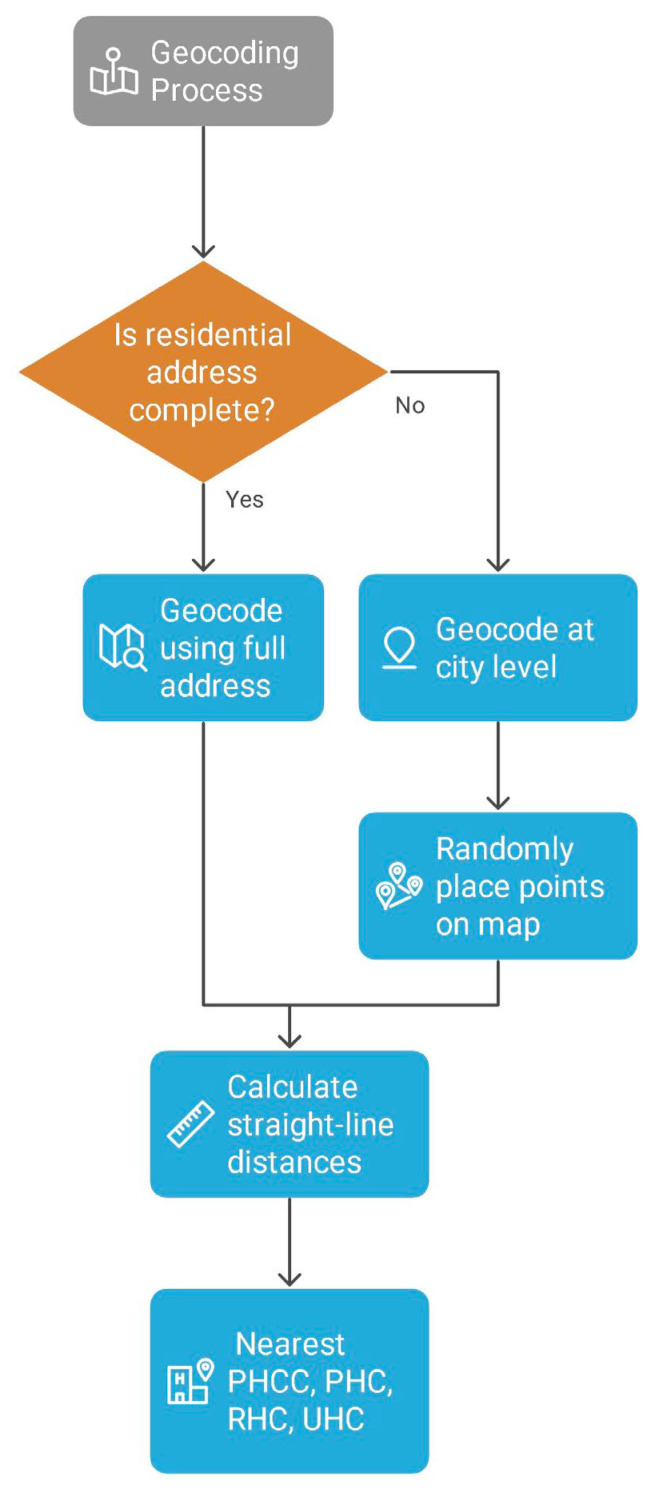
Geocoding process.

The locations of public healthcare facilities were geocoded accurately at the address plate and provided by the Ministry of Health, including PHCCs, PHCs, RHC, and UHC.The geocoding process aimed to pinpoint the most accurate locations of the healthcare facilities based on available address details. Participants addresses were geocoded by a third-party enterprise using the addresses on the ID cards, and if this information wasnot available, geocoding was performed with the addresses provided by the participants to the secretary. The accuracy of the geocoding varied based on the level of available detail for the addresses. Addresses were geocoded according to administrative and statistical criteria established by the High Commission for Planning of Morocco, using douars (rural administrative units) and rural communes. Urban patients were geocoded using full residential addresses, streets, subneighborhoods, neighborhoods, and municipalities. In cases where this information was incomplete; geocoding was performedat the city level, with points placed randomly on the map. To determine travel distances, straight-line (Euclidean) distances in kilometers were calculated from women’s residences to the nearest PHCC, PHC, RHC, or UHC. For geographic analysis, ArcMap 10.8 software [21] was used to calculate distance and visualize the distribution of cancer cases in the Casablanca-Settat region (Figure 2).

### 2.5. Ethical Consideration

This study was conducted according to ethical guidelines and standards for research involving human participants. Ethical approval was obtained from the ethics committee of Tangier University Hospital under the reference number AC97JT/2024. Participants’ confidentiality and privacy were strictly maintained throughout the study.

### 2.6. Statistical Analyses

Descriptive statistics were used to summarize patient characteristics, including age, residence, cancer stage, and travel distance to healthcare facilities. Chi-square (χ^2^) tests were performed to assess the association between urban and rural residences and breast cancer stages. The Mann–Whitney U test was used to compare the median travel distances to the UHC, RHC, PHCs, and PHCCs between early stages and advanced cancer stages, as this non-parametric test does not assume a normal distribution of the data. A *p*-value < 0.05 was considered statistically significant. All analyses were conducted using SPSS 21.0.

To assess the robustness of our findings, sensitivity analysis was performed using only participants with high geocoding accuracy. Results were compared with the main analysis to evaluate consistency and to identify potential biases or influential factors.

## 3. Results

### 3.1. Characteristics of the Study Population

**Figure 3:**
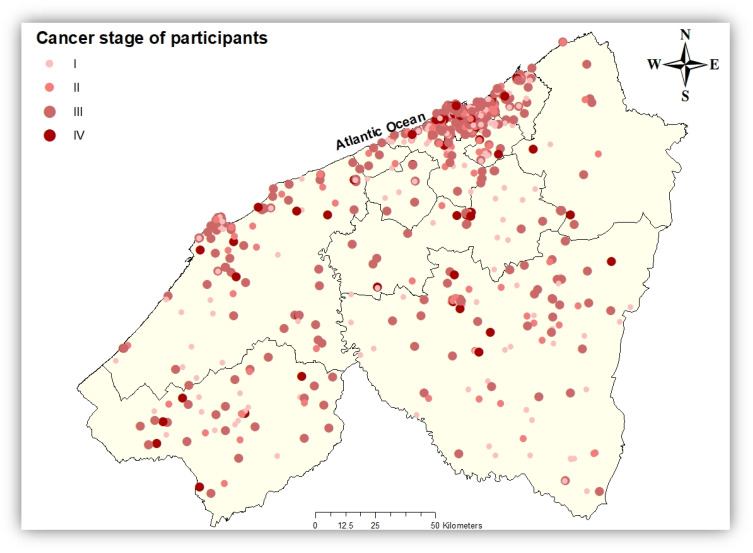
Spatial distribution of breast cancer cases with known cancer stage in the Casablanca-Settat region at diagnosis.

From December 2018 to January 2022, 2161 women with breast cancer residing in the Casablanca-Settat region were admitted tothe university hospital center of Ibn Rochd, with 27.6% of them living in the rural area of the region. The study population is predominantly middle-aged, with 61.1% aged between 40 and 59 years. Regarding Body Mass Index (BMI), 49.3% of the population had a normal weight (BMI 18.5–24.9), 32.0% were classified as overweight (BMI 25–29.9), and 18.7% were obese (BMI >30). Health coverage data revealed that 89% of participants were covered bythe RAMED. Among participants with available cancer stage information, 38% were classified as Stage III, and 8.5% were classified as Stage IV (Figure 3).

### 3.2. Distribution of Breast Cancer Cases

The distribution of breast cancer cases varied across provinces in the Casablanca-Settat region. Casablanca recorded the highest number of cases (*n* = 1264; 58.5%). Other provinces with notable case counts included El Jadida (*n* = 2.25; 10.4%) and Settat (*n* = 212; 9.8%). Mohammedia reported 126 cases (5.8%), followed by Berrechid (177 cases;8.2%) and Sidi Bennour (*n* = 77;3.6%). The provinces of Nouaceur (*n* = 42; 1.9%), Benslimane (*n* = 24; 1.1%), and Mediouna (*n* = 14; 0.6%) reported smaller numbers of cases (Table 1).

### 3.3. Access to Healthcare Facilities

**Figure 4:**
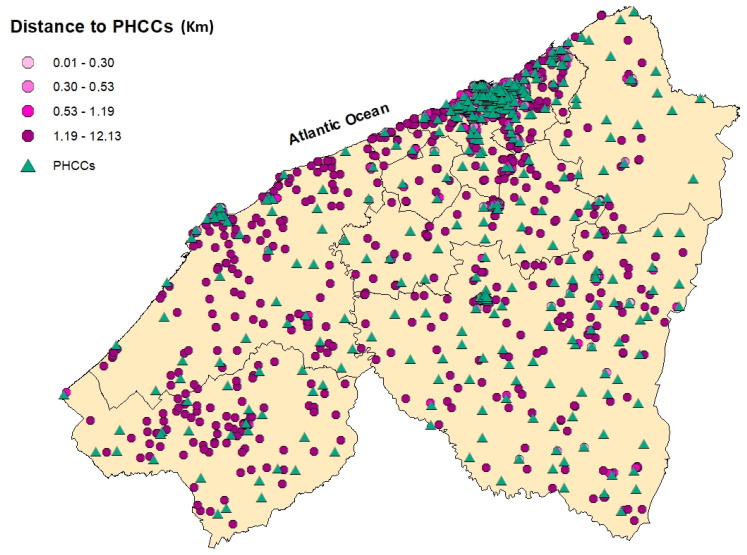
Spatial distribution of breast cancer cases in the Casablanca-Settat region according to their travel distance to the nearest Primary Healthcare Center (PHCC). The distances are categorized by quartiles: 0.01–0.30 km (lightest pink), 0.30–0.53 km (medium pink), 0.53–1.19 km (bright pink (mafenta)), and 1.19–12.13 km (dark purple).

**Figure 5:**
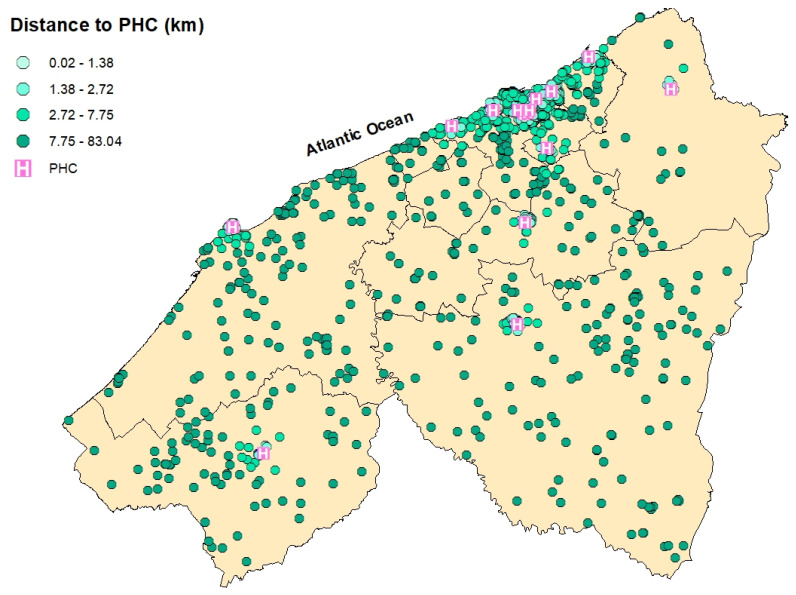
Spatial distribution of breast cancer cases in the Casablanca-Settat region according to their travel distance to the nearest Provincial Hospital Center (PHC). The distances are categorized by quartiles: 0.02–1.38 km (very light teal), 1.38–2.72 km (light teal), 2.72–7.75 km (Bright Teal), and 7.75–83.04 km (green).

**Figure 6:**
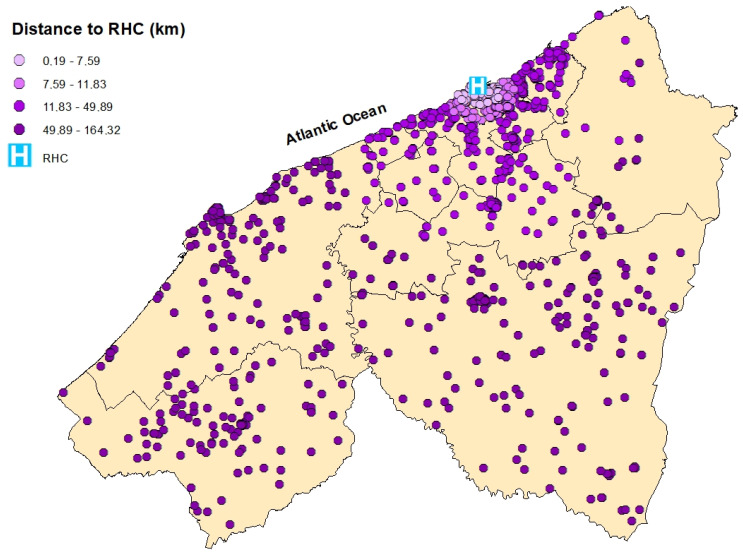
Spatial distribution of breast cancer cases in the Casablanca-Settat region according to their travel distance to the Regional Hospital Center (RHC). The distances are categorized by quartiles: 0.19–7.59 km (very light purple), 7.59–11.83 km (light purple), 11.83–49.89 km (bright purple), and 49.89–164.32 km (dark purple).

**Figure 7:**
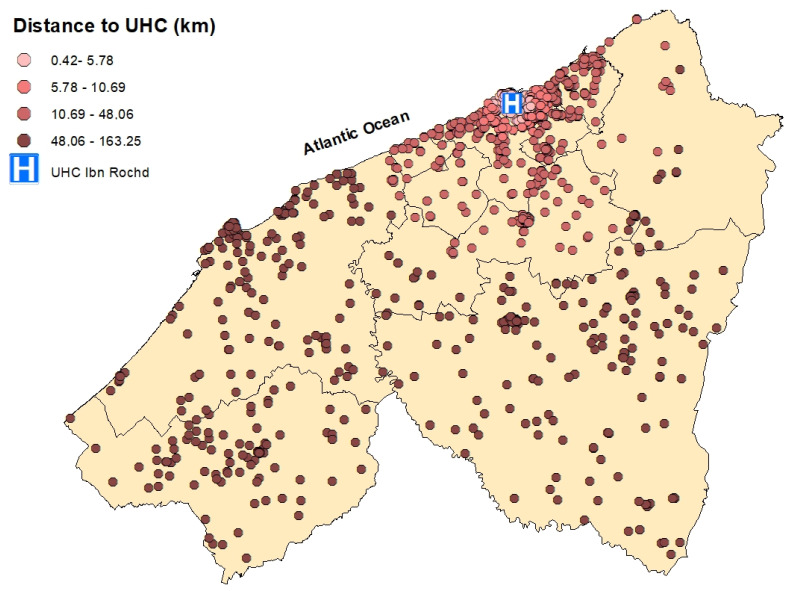
Spatial distribution of breast cancer cases in the Casablanca-Settat region according to their travel distance to the University Hospital Center (UHC) Ibn Rochd. The distances are categorized by quartiles: 0.42–19.94 km (very light pink), 19.94–55.20 km (rosy pink), 55.20–103.95 km (light brown), and 103.95–163.25 km (dark brown).

Table 2 and Figure 3, Figure 4, Figure 5, Figure 6 and Figure 7 summarizedistances (in km) to different healthcare facilities—UHC, RHC, PHCs, and PHCCs—by urban and rural areas.

Overall, rural participants consistently reported greater distances to health facilities compared to participants residing in urban areas. The median distance to the UHC was 7.27 km in urban areas and 63.3 km in rural areas, with an overall median of 10.7 km. Similarly, the median distance to the RHC was 9.13 km for urban participants and 64.4 km in rural settings (overall: 12.3 km). Access to PHCs was relatively better, with a median distance of 1.88 km in urban areas and 17.8 km in rural areas (overall: 2.81 km). However, PHCCs were the most accessible in both settings, with a median of 0.41 km in urban areas and 2.75 km in rural areas (overall: 0.53 km).

### 3.4. Association Between Proximity to Healthcare Facilities, Area of Residence, and Cancer Stage

Table 3 presents the association between cancer stage, proximity to healthcare facilities, and area of residence (urban vs. rural). No statistically significant association was observed between cancer stage and area of residence (χ^2^ = 1.62, *p* = 0.8), indicating that the distribution of advanced and early cancer stages was similar in both urban and rural populations. Similarly, no statistically significant difference between patients diagnosed at early vs. advanced stages was observed in the distance to the UHC (U = 0.01, *p* =0.53), RHC (U= 0.04, *p* = 0.49), PHCs (U = 0.09, *p* = 0, 14), and PHCCs (U = 0.9, *p* = 0, 55).The sensitivity analysis reported results consistent with the primary analysis, and the overall conclusions remained unchanged.

## 4. Discussion

This is the first national study that examines the distribution of breast cancer cases in the region of Casablanca-Settat and considers access to healthcare facilities among women with breast cancer admitted to the university hospital center of Casablanca, as it is the first to evaluate the association between residential environment (urban/rural) and breast cancer stage.

**Figure 8:**
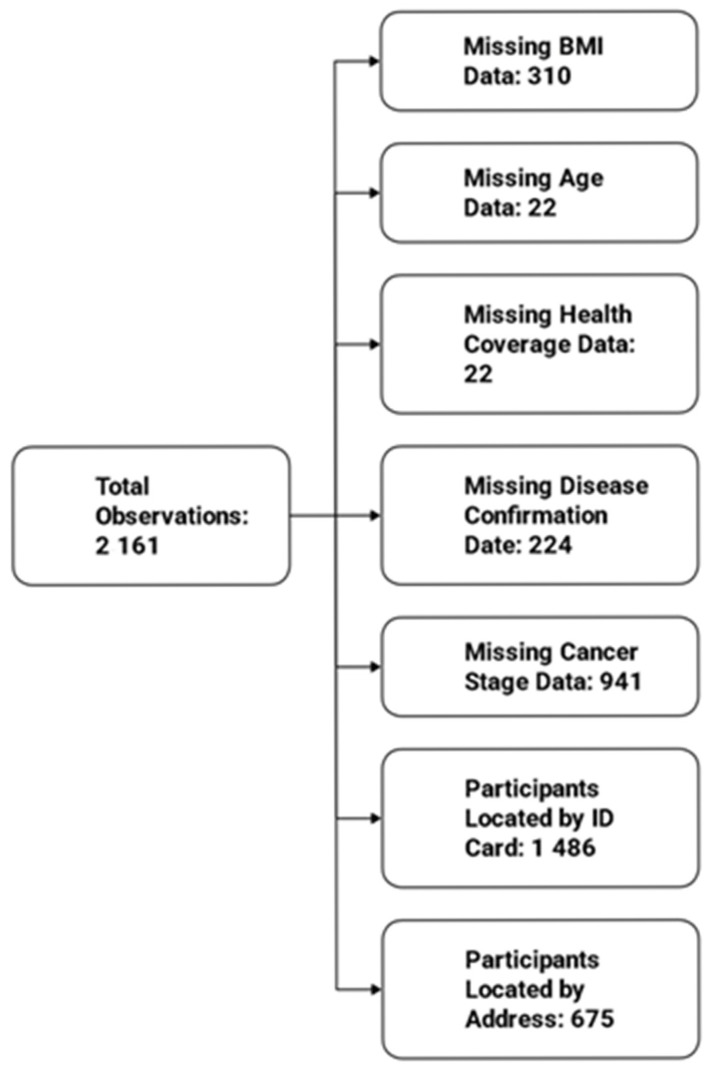
Missing Data.

In total, out of2161 observations, 310 participants were missing data for BMI, 22 were missing age information, 22 were missing health coverage, 224 participants lacked the date of disease confirmation, and 941 had missing cancer stage. Additionally, 1486 participants were located based on ID card residence, while 675 lacked this information and were located using addresses provided to the secretary (Figure 8).

This study revealed that 58.5% of women with breast cancer were residing in Casablanca, highlighting the concentration of cases in this major urban center. This finding is consistent with Casablanca’s status as Morocco’s most populous city and its role as the region’s economic and healthcare hub. Also, for the675 breast cancer cases that lacked recorded ID card addresses, we relied instead on addresses provided to the secretary. Among these, 201 women reported living in Casablanca. It is strongly hypothesized that many participants from remote areas or other distant cities without cancer treatment centers might have used the address of a relative or friend living near the health facilities, where they stayed during the treatment period [21], which may have led to an overestimation of cancer cases recorded in the city of Casablanca.

The study population is predominantly covered by RAMED health coverage (89%), highlighting their socioeconomic vulnerability. These participants rely solely on public health facilities. In contrast, participants with CNOPS or CNSS health coverage may have better socioeconomic status as these health coverage systems typically apply to individuals with stable jobs in the public or private sector which provide regular income in the household. Since CNOPS and CNSS also cover services in the private sector, it is possible that patients with higher incomes may prefer private hospitals and cancer centers to avoid delays and waiting times. This could explain the relatively small percentage of individuals with CNOPS and CNSS health coverage admitted in the UHC in our study population (8.43%). These findings are in line with a study conducted by Zakeriet al. in Iran on the use of health services according to socioeconomic status and health coverage [22]. The authors revealed that individuals with complementary insurance use private services more. Similarly, Bourne et al. reported comparable results in Jamaica [23]. Individuals in the wealthiest income quintile are significantly more likely to access private healthcare. However, those in the poorest quintiles use predominantly public healthcare services, where costs are lower and more affordable for those with limited financial resources [23].

The analysis of distances to healthcare facilities across urban and rural settings highlights substantial geographic inequalities in physical access to care. Interestingly, PHCs and PHCCs were more accessible, even in rural zones. The median rural distance to PHCCs was only 2.75 km and 17.8 km for PHCs, indicating that basic health services are within reach for most participants. In Morocco, PHCCs are intentionally distributed as the first point of contact in the national healthcare system [24].Their placement is guided by the Ministry of Health based on population density and geographic need to ensure equitable access to primary care [24]. The difference in distance was pronounced for higher-tier facilities, for instance, the median distance to the UHC in rural areas is 63.3 km, compared to only 7.27 km in urban zones, and 64.4 km for the RHC versus 9.13 km in urban areas. These long distances are particularly meaningful: a distance of over 60 km likely represents more than one hour of travel by car, potentially posing a serious barrier for individuals with limited transport means or financial constraints. However, no statistically significant association was found between distances to the UHC or RHC and cancer stage at diagnosis, suggesting that physical distance alone may not fully explain delays in diagnosis. Other barriers include limited transportation options [25,26], highcosts [27], lack of awareness and knowledge regarding breast cancer [27], cultural beliefs [26], fear of diagnosis [28], or delays within the health system [29].

Our findings are in line with several studies that also reported no association between rural residency, travel distance, and cancer stage mainly in developed countries [11,12,13,14]. For instance, a study by Celaya et al., which analyzed data from New Hampshire (1998–2005), found no significant rural–urban difference in the stage of breast cancer at diagnosis [30]. The authors attributed this lack of association to the absence of detailed mammography data, which could have provided a clearer picture of screening access and its potential impact on early cancer detection. Similarly, Henry et al., using data from a 10-state cancer registry in the US, found differences in cancer stage, suggesting that living in close proximity to a diagnosing facility does not ensure better health outcomes; and other non-geographic factors such as poverty, race/ethnicity, and health insurance independently present more substantial risks for a late-stage diagnosis of breast cancer [31]. In contrast, many studies found associations between travel distance, rural residence, and cancer stage. For example, a meta-analysis including 21 studies [13] (USA, New Zealand, Australia, Denmark, South Africa, Egypt, Poland, Italy), with a combined sample size of 879,660 women, indicated that rural women had 1.19 times higher odds (95%confidence interval: 1.12–1.27) of being diagnosed at a later stage of breast cancer compared to urban women. Another systematic review and meta-analysis from East Africareportedthatwomen living in rural areas weresignificantly more likely to present at an advanced stage comparedwithwomen living in urban areas [32]. Similarly, a systematic review and meta-analysis in Ethiopia found that the prevalence of advanced-stage breast cancer diagnosis was 72.56%with an AOR of 2.04, indicating that rural residents had more than twice the odds of being diagnosed at an advanced stage compared to urban residents [33].Also, in Sub-Saharan Africa, studies showed a significant association between travel distance and cancer outcomes [34]. Togawa et al. [34] found that, for every additional 50 km traveled, the odds of a late-stage diagnosis increase by 4%. Similarly, Tesfaw et al. [35] in Ethiopia reported that breast cancer patients traveling 5 km or more to the nearest healthcare facility were significantly more likely to be diagnosed at an advancedstage (AOR = 3.2; 95% CI: 1.72, 5.29).

Although the existing literature on the impact of rural–urban disparities and travel distance on the stage at breast cancer diagnosis remainsheterogeneous, it is important to consider factors that might strengthen this association. For instance, socioeconomic status could play a key role by influencing access to healthcare, screening behaviors, and diagnostic delays.

The study has certain limitations, notably the percentage of missing data regarding cancer stage (approximately 43.5%), which may affect the interpretation of the results. The incidence rate of breast cancer in the region was not provided in this study due to the lack of information on the number of new cases in the private sector. Furthermore, our sample focused exclusively on patients admitted to public hospitals, reflecting a more economically vulnerable population but limiting the generalizability of the findings, as approximately 56% of breast cancer patients treated in private clinics are not included in our study [36]. We encourage future research to include patients from the private sector to improve representativeness and provide a more comprehensive understanding of breast cancer patterns across different socioeconomic groups. Also, the design of our study did not capture information on education, household roles, cultural beliefs, transportation availability, or health literacy. Future studies could address these limitations by adopting a prospective design. Additionally, the implementation of standardized data collection protocols and the use of electronic health records could further strengthen data quality in such studies.

## 5. Conclusions

This study provides valuable insights into the distribution of breast cancer cases and the potential influence of geographic factors on cancer stage among women in the Casablanca-Settat region. Notably, our findings indicate that rural residence and geographic proximity to healthcare facilities do not significantly impact the stage of breast cancer at diagnosis. However, barriers such as limited transportation, socioeconomic status, and delays in admission may still impact timely access. Further research is needed to explore the impact of social determinants of health, patient health-seeking behavior, and systemic healthcare delays to better inform targeted interventions aimed at promoting earlier diagnosis and improving cancer care equity in Morocco.

## Figures and Tables

**Table 1:** Demographic characteristics of the study population.

		(N)	(%)
Age	<40	204	9.5 %
	40–49	615	28.7 %
	50–59	694	32.4 %
	60–70	419	19.6 %
	70–79	161	7.5 %
	≥80	46	2.1 %
	NA	22	-
BMI	Normal (−24.9)	912	45.4%
	Overweight (25–29.5)	593	29.5%
	Obesity (+30)	346	17.2%
	NA	310	-
Cancer stage	I	385	31.6 %
	II	38	3.1 %
	IIA	61	5.0 %
	IIB	168	13.8 %
	III	269	22.0 %
	IIIA	59	4.8 %
	IIIB	135	11.1 %
	IIIC	1	0.1 %
	IV	104	8.5 %
	NA	941	-
Health coverage	Ramed	1924	89.0%
	Private	154	7.1%
	Public	28	1.3%
	Other	55	2.5%
	NA	22	-
Province/ Prefecture	Benslimane	24	1.1%
	Berrechid	177	8.5%
	Casablanca	1264	58.5%
	El jadida	225	10.4%
	Mediouna	14	0.6%
	Mohammedia	126	5.8%
	Nouaceur	42	1.9%
	Settat	212	9.8%
	Sidi bennour	77	3.5%
Area of residence	Rural area	596	27.6%
	Urban area	1565	72.4%

**Table 2:** Geographic Accessibility ofDifferent Levels of Health Facilities by Residence Type in the Casablanca-Settat Region.

Facility Type	Distance to UHC			Distance to RHC			Distance to PHCs			Distance to PHCCs		
	Urban	Rural	Overall	Urban	Rural	Overall	Urban	Rural	Overall	Urban	Rural	Overall
Median distance (km)	7.27	63.30	10.7	9.13	64.40	12.3	1.88	17.8	2.81	0.41	2.75	0.53
The interquartile mean	8.59	74.90	42.3	8.79	74.90	42.3	2.43	19.4	10.6	0.38	3.27	0.88
Minimum distance (km)	0.42	9.80	0.42	0.42	13.30	0.192	0.19	5.43	0.02	0.02	0.08	0.01
Maximum distance (km)	161	163	163	162	164	164	67.3	83.0	83.0	4.61	12.1	12.1

**Table 3:** The association between cancer stage, proximity to healthcare facilities, and residence type (urban vs. rural).

		Early Cancer Stage		Advanced Cancer Stage		*p*-Value
		Median	IQR	Median	IQR	
Distance to PHCCs (km)		0.52	0.30–1.04	0.54	0.30–1.25	0.55
Distance to PHCs (km)		2.53	1.37–6.45	2.81	1.44–8.04	0.14
Distance to RHC (km)		11.35	7.76–46.29	11.09	7.29–51.66	0.49
Distance to UHC (km)		10.22	5.85–44.52	9.91	5.65–49.03	0.53
		N	%	N	%	*p*-Value
Area of residence	Rural residence	165	13.52%	163	13.36%	0.81
	Urban residence	487	39.92%	405	33.20%	

## Data Availability

The data presented in this study are available on request from the corresponding author.

## Abbreviations

The following abbreviations are used in this manuscript:

AOR	Adjusted odds ratio
BMI	Body mass index
CNOPS	Caisse Nationale des Organismes de Prévoyance Sociale (Public health insurance for civil servants and public-sector employees in Morocco)
CNSS	Caisse Nationale de Sécurité Sociale (Private health insurance for private-sector employees in Morocco)
ENOVA	Electronicmedical system used at Ibn Rochd University Hospital
GLOBOCAN	Global Cancer Observatory
PHCC	Primary healthcare center
PHC	Provincial hospital center
RAMED	Régime d’Assistance Médicale (Morocco’s program offering free healthcare services to vulnerable populations)
RHC	Regional hospital center
TNM	Tumor, node, metastasis classification system
UHC	University hospital center
